# Carbon-Based Nanomaterials via Heterojunction Serving as Photocatalyst

**DOI:** 10.3389/fchem.2019.00713

**Published:** 2019-10-24

**Authors:** Noureen Syed, Jianfeng Huang, Yongqiang Feng, Xiao Wang, Liyun Cao

**Affiliations:** Shaanxi Key Laboratory of Green Preparation and Functionalization for Inorganic Materials, Key Laboratory of Auxiliary Chemistry and Technology for Chemical Industry, School of Materials Science and Engineering, Ministry of Education, Shaanxi University of Science and Technology, Xi'an, China

**Keywords:** carbon nanomaterials (CNMs), photocatalysts, heterojunction, water splitting, hydrogen production

## Abstract

Photocatalytic hydrogen production from water splitting is of auspicious possibility to resolve the energy shortage and environmental anxieties. In the past decade, the combination of different carbon-based allotropes with semiconductors of different structure and unique properties to construct heterojunction, which can improve the charge separation, light absorption, and steadiness, offer a promising way to achieve efficient photocatalyst. This review aims to provide an overview of the development for the carbon nanomaterials (CNMs)-based photocatalysts used for hydrogen production from water splitting and photocatalytic degradation of organic pollutants in waste water. The recent progress of CNMs-based heterojunction, including various composite with graphene, fullerene, carbon quantum dots (CQDs), and carbon nanotubes (CNTs) were highlighted. Furthermore, a typical model of CNMs-based Z-scheme heterojunction was also addressed. Finally, a promising perspective on the future development of CNMs-based photocatalysts have been discussed.

## Introduction

Photocatalysts facing toward energy crisis and environmental issues have attracted increased intention as one of the best way for the reduction of toxic contaminants and H_2_ production (Hisatomi et al., 2014; Low et al., 2015; Dai et al., 2017; Liu G. et al., 2019). However, challenges for the photocatalysts remains regarding to the limited light absorption, high charge recombination, and low quantum yield (Sudhaik et al., 2018). Up to now various photocatalysts have been developed to resolve these issues, among which carbon-based photocatalysts recently aroused tremendous interest due to their large surface area, favorable electronic conductivity, low fabrication cost, and high chemical/thermal stability (Yang et al., 2014; Xia et al., 2017; Ma et al., 2018). These unique properties make carbon nanomaterials (CNMs) as the most promising candidate for photocatalysts (Yu et al., 2014).

The most widely used CNMs for the synthesis of photocatalysts, such as graphene (Yu et al., 2016), carbon nanotubes (CNTs) (Zhang Y. et al., 2019), carbon quantum dots (CQDs) (Li Y. et al., 2018), fullerene (Song et al., 2017), and graphitic carbon nitride (g-C_3_N_4_) (Zhang S. et al., 2019) have attracted great attention due to their high physiochemical stability, earth abundant, and low synthesis cost. Moreover, the electronic structure and photocatalytic properties of CNMs could be adjusted through morphology and interfacial modulation (Xin et al., 2018). Whereas, pristine CNMs suffer from rapid recombination of electron-hole pair and narrow visible light adsorption. One of the best strategy to solve this problem is to construct heterojunction via assembly of CNMs with semiconductors. Especially, the modified CNMs-based Z-scheme heterojunction, resembling the natural photosynthetic model, benefit from various merits including improved light harvesting, spatially separated electron and hole sites and strong redox ability (Tong et al., 2012). Beside the structure modification of the Z-scheme heterojunction, the introduced CNMs also serve as electron mediator between two semiconductors, which actually reduce the resistance and improve the charge separation and stability.

This paper aims to provide an overview of carbon-based photocatalysts in water splitting for H_2_ production as well as degradation of organic pollutants. The properties, performances, and combinations of different allotropes of carbon as photocatalysts were discussed. Photocatalytic enhancements by solid Z-scheme heterojunction were also reviewed.

## Carbon-Based Photocatalysts

### Graphene as Photocatalyst

Graphene with excellent physical and chemical properties discovered in 2004, holding sp^2^-hybridized atoms tightly assembled into an ordered two-dimensional (2D) honeycomb construct, offer new opportunities in designing efficient photocatalytic materials with high stability (Gupta et al., 2019; Madkour, 2019). Recent demand for the synthesis of metal-free photocatalysts is on the verge of increase. Gong et al. successfully obtained graphene/g-C_3_N_4_ nanocomposites by impregnation chemical reduction strategy, which served as active photocatalysts for H_2_ production in visible light (Gong et al., 2018). Moreover, 2D graphene and transition metal dichalcogenides (TMDCs), have become versatile nanomaterials for the fast progress of photocatalysts due to their unique properties in optical, electrical, thermal, and mechanical aspects (Rosman et al., 2018). One of the works done by Lv et al. that elaborated graphene composite without noble metal, revealed that graphene attached to semiconductor surface fabricated by hydrothermal method can efficiently accommodate and transport electrons from the excited semiconductor, which not only hindered charge recombination but also improved charge transfer, giving rise to high photocatalytic efficiency (Lv et al., 2012). This work confirmed the significant contribution of graphene in enhancing the photocatalytic activity. Afterwards, many graphene-based photocatalysts have been developed. For example, Quiroz-Cardoso et al. (2019) recently reported graphene in combination with nickel nanoparticles modified CdS fibers (Ni/GO-CdS) enhanced the photocatalytic hydrogen production, which was 6.3 times higher than that of bare CdS. Considering the superior conductivity and tunable structure, graphene would be the most promising candidate for photocatalysts. Design and construction of novel hierarchical architectures hybridizing with graphene nanostructures would provide plenty of rooms for photocatalytic application.

### CNTs as Photocatalyst

Photocatalytic water-splitting technology based on CNTs-modified nanomaterials has exhibited great potential for hydrogen production in view of their low cost and high stability (Yi et al., 2018). For example, Zheng et al. (2008) has offered new opportunities for achieving high photocatalytic activity with high stability. In combination of CNTs with graphene not only increase reaction sites but also inhibit the recombination of photo-excited electron-hole pairs (Bhanvase et al., 2017). In addition, CNTs-based photocatalysts also revealed high activity on the photocatalytic degradation of organic pollutants due to π-system or formation of heterojunction. For example, CNT-modified hierarchical microspheres ZnO enhanced the visible light adsorption and charge separation process, exhibiting excellent photocatalytic performance much better than the pure ZnO for the reduction of the organic molecules in the industrial effluents (Ahmad et al., 2014). Combination of CNT with other photocatalysts could enhance the conductivity and facilitate the charge transfer process during the photocatalytic reaction. To further improve the performance in future, more efforts should be made to *in-situ* synthesize CNT-based composite in order to strengthen the synergetic interaction between CNT and other nanostructures.

### CQDs as Photocatalyst

CQDs as an emerging and recently developed CNMs provided well-controlled intrinsic characteristics because of its unique optical and electrical properties, as well as the special fluorescence emission feature (Zhang et al., 2017). Since their discovery in 2004 (Xu et al., 2004), CQDs have been utilized in various application, including chemical sensor, bioimaging, nanomedicine, photocatalysts, etc. Particularly, in photocatalytic application, CQDs showed the most promising potential for photocatalytic H_2_ production. Moreover, CQDs can act both as electron acceptor and donor leading to effective electron and hole separation, and extensively modify the photo-absorption range of semiconductor materials with large band gap to visible regions (Pirsaheb et al., 2018). Wang et al. demonstrated that metal-doped CQDs combined with CdS nanowires as a co-catalyst showed much better hydrogen production performance than the undoped CQDs/CdS composite (Wang Y. et al., 2019). One more example examined by Wang et al. demonstrated that the visible-light-sensitive BiVO_4_ quantum tube (q-BiVO_4_) decorated with CQDs displayed outstanding photocatalytic performance, whose kinetic constants for the degradation of phenol and rhodamine B (RhB) were 3.0 and 2.4 times higher than those of the sole q-BiVO_4_, respectively (Wang G. et al., 2019). Due to the potential both as electron donor and acceptor, CQD should be further investigated in the field of photocatalytic application. Developing novel and facile green synthesis method to fabricate CQD-based CNMs, especially the metal-free catalysts deserves more attention.

### Fullerene as Photocatalyst

Fullerene (C_60_) with a close-shell shape consisting of 20 hexagons and 12 pentagons, holding 30 orbital bonding with 60 p-electrons, has been recognized as the most significant carbon allotropes because of the unique chemical and physical characteristics (Lindqvist et al., 2014). Besides, C_60_ is both an excellent electron acceptor and donor, which facilitate the functionality of fullerene-based carbon materials in photocatalytic applications. Encapsulation of fullerene into CNTs is super effective technique, pioneered by Smith et al. (1998), to fabricate heterojunction with unique electronic characteristics (Rahimi-Nasrabadi et al., 2017). Song et al. synthesized a novel C_60_/graphene/g-C_3_N_4_ composite with high hydrogen production efficiency for water splitting (Song et al., 2017). The synergetic effect between graphene and C_60_ improved the transportation and utilization efficiency of photo-generated electrons and accelerated the separation of photo-generated electron and hole pairs, thus considerably enhancing the hydrogen generation ability of g-C_3_N_4_. Fullerene and its derivatives have been widely used in the organic photovoltaic device, however, their application in photocatalysis for hydrogen production and organic pollutants degradation is in the infancy. Until now, most works only focused on the C_60_, other members in fullerene family such as C_70_ and their derivatives should be pay more attention for photocatalysts in future.

### g-C_3_N_4_ as Photocatalyst

Recently, the improvement of photocatalytic activity by using g-C_3_N_4_ has turned into a hot research subject because of its tunable electronic band structure, highly stable physiochemical properties, simple manufacturing and low cost (Dong et al., 2016, 2019; Li Y. et al., 2018). Wang J. et al. (2019) fabricated a 3D flower-like TiO_2_ hybridized with 2D g-C_3_N_4_ nanosheet through a hydrothermal and calcination process. The resulting TiO_2_/g-C_3_N_4_ composite exhibited a much enhanced efficiency of photocatalytic hydrogen production, which is 7.7 and 1.9 times higher than that of the pure g-C_3_N_4_ and TiO_2_, respectively. It was reported that the extended visible light adsorption by g-C_3_N_4_ make a contribution to the improved photocatalytic performance. In addition, the activity of g-C_3_N_4_ could also be improved by doping. Zhou et al. (2019) reported that the NO removal rate of g-C_3_N_4_ could be enhanced by 1.5 times after Sr doping. Density functional theory (DFT) method is powerful for systematically depicting the electronic structures and understanding energy-related mechanism for photocatalytic reaction. The results revealed that different doping modes of Sr including intercalation, cavity padding, replacement of triazine N and bridging N could decrease the band gap of g-C_3_N_4_, thus facilitating the charge transfer process.

## Heterojunction

**Figure 1:**
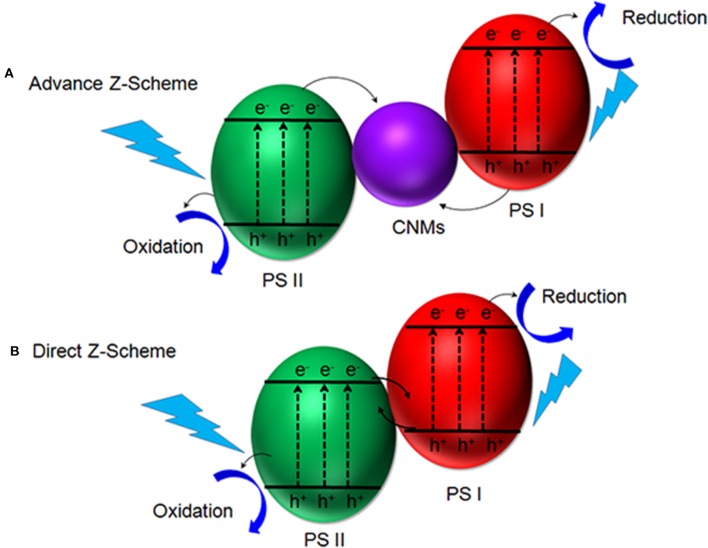
Schematic illustrations of an advanced Z-scheme heterojunction with CNMs as an electron mediator **(A)** and the direct Z-scheme heterojunction **(B)**. Modified from Li H. et al. (2015). with copyright permission from John Wiley and Sons, Inc.

Many efforts have been made to realize the complete utilization of photo-excited charge carriers and inhibit recombination of electron-hole pairs during the photocatalytic process, among which fabrication of heterojunction is one of the best approach to improve the charge separation efficiency and reduce the recombination of the photogenerated electron-hole pairs (Moniz et al., 2015; Wang et al., 2018). The most commonly-investigated heterojunction is constructed via two solid semiconductors and one electron mediator as illustrated in Figure 1, forming an advanced solid-type Z-scheme configuration. The main mechanism for Z-scheme heterojunction is inspired by the natural photosynthesis process. Electrons of photocatalysts I are recombined with holes in photocatalysts II via electron mediator under light, which inhibits the recombination of photo-induced charge carrier and stay their redox property (Bard and Fox, 1995).

In order to improve the conductivity of semiconductor photocatalysts, CNMs like graphene, fullerene, CNTs and their derivatives have been widely used in different heterojunction as an electron mediator to increase the conductivity (Natarajan et al., 2018). Table 1 summarize several typical carbon-based photocatalysts. As compared with other carbon-based heterojunction photocatalysts, graphene exhibits a plenty of merits such as low cost, large surface area and tunable band structure (Li X. et al., 2018). Gebreslassie et al. demonstrated that graphene as an electron mediator can significantly enhance the photocatalytic activity, which showed 7–15-folds higher H_2_ production compared with their pristine compounds without the aid of graphene (Gebreslassie et al., 2019). Similarly, Jiang et al. synthesized a solid-state Z-scheme Bi_2_WO_6_/CNTs/g-C_3_N_4_ composite, where CNT acted as an electron mediator (Jiang et al., 2018). This composite disclosed an outperforming photocatalytic activity than the pure Bi_2_WO_6_ and g-C_3_N_4_ for the degradation of 2,4-dibromophenol. Recently, CQDs have also been used as electron mediator to build solid-state Z-scheme heterojunction. In 2019, Liu et al. fabricated a CQD-based Z-scheme heterojunction by bridging TiO_2_ and Cd_0.5_Zn_0.5_S with CQDs, which exhibits super photocatalytic activity for H_2_ evolution (Liu E. et al., 2019). Meanwhile, Pan et al. constructed a sandwich-type structure, where CQDs were embedded between CdS and BiOCl (Pan et al., 2018). The resulting CdS/CQDs/BiOCl heterojunction displayed much higher photocatalytic activity on the degradation of RhB and phenol under visible and UV light illumination compared with BiOCl, CdS/BiOCl, and CQDs/BiOCl.

**Table 1:** Summary of carbon-based photocatalysts.

**Photocatalysts**	**Heterojunction type**	**Synthesis method**	**References**
PPTA/MWNTs	N.A.^a^	Polycondensation	Mazrouaa et al., 2019
g-C_3_N_4_/graphene/NiFe_2_O_4_	Solid state Z-scheme	Hydrothermal	Gebreslassie et al., 2019
CN/CNT/BWO	Solid state Z-scheme	N.A.	Jiang et al., 2018
Bi_2_WO_6_/g-C_3_N_4_	Direct Z-scheme	Hydrothermal	Li M. et al., 2015
ZnO/g-C_3_N_4_	Direct Z-scheme	Solid state	Yu et al., 2015
Cd_0.5_Zn_0.5_S/CQD/TiO_2_	Solid state Z-scheme	Hydrothermal	Liu E. et al., 2019
Cds/CQDs/BiOCl	Solid state Z-scheme	Facile-region	Pan et al., 2018
Ru/SrTiO_3_	Z-scheme	Hummers method	Iwase et al., 2011
SnS_2_/g-C_3_N_4_	Z-scheme	Hydrothermal	Di et al., 2017
SnO_2−x_/g-C_3_N_4_	Z-scheme	Solid-state synthesis	He et al., 2015
CdS/SiC	Z-scheme	Hydrothermal	Peng et al., 2015
CdS/graphene	N.A.	N.A.	Li et al., 2011
ZnIn_2_S_4_/RGO	N.A.	Solvothermal	Ye et al., 2014
Bi_2_WO_6_/graphene	N.A.	Sonochemical	Sun et al., 2014
Graphene/g-C_3_N_4_	N.A.	Impregnation–chemical reduction	Xiang et al., 2011
Nanoparticle/graphene	N.A.	One-pot solution	Lv et al., 2012
TiO_2_/graphene	N.A.	Sol gel method	Zhang et al., 2010
TiO_2_/carbon dots	N.A.	Hydrothermal	Wang et al., 2014
CdS/graphene	N.A.	Hydrothermal	Ye et al., 2012
Ta_2_O_5_/CNT	Schottky heterojunction	N.A.	Cherevan et al., 2014
Ni/GO-CdS	N.A.	Photo-deposition	Quiroz-Cardoso et al., 2019
La-CNTs/TiO_2_	N.A.	Sol-gel method	Tahir, 2019
TiO_2_/CQD	N.A.	Green synthesis	Sargin et al., 2019

a *N.A., Not Available*.

## Conclusions and Perspectives

Carbon-based nanomaterials with low cost and favorable catalytic performance have been extensively used for photocatalytic reactions in the fields of energy conversion and environmental protection. In this review, CNMs such as graphene, CNTs, CQDs, C_60_, and g-C_3_N_4_, etc. used as photocatalysts in the application for H_2_ production from water splitting and photocatalytic degradation of organic pollutants in waste water were comprehensively overviewed. It could be concluded that CNMs exhibit intriguing property in enhancing the photocatalytic performance of various photocatalysts. With the rapid development of advanced technique, various carbon-based Z-scheme heterojunction with excellent photocatalytic performance have been established. This type of heterojunction inspired by artificial photosynthesis method possess many advantages including increased light harvesting and favorable strong redox capability, which highly improved the photocatalytic performance compared with the direct heterojunction. Different type of carbon allotropes are the good performer as an electron mediator in solid-state Z-scheme heterojunction while the selection of proper electron mediator with specific composite to different materials according to their specific function is challenging and crucial. Obviously photocatalytic efficiency depends on the type of material. The development of novel photocatalysts with better catalytic performance has always been put forward to the frontiers of nanomaterials and a further understanding of heterojunction mechanisms are also of great importance to promote the application of photocatalysts.

## Author Contributions

NS and YF organized and wrote the manuscript. XW, JH, and LC discussed the results and revised the paper. All authors approved this publication.

### Conflict of Interest

The authors declare that the research was conducted in the absence of any commercial or financial relationships that could be construed as a potential conflict of interest. The reviewer DG declared a shared affiliation, with no collaboration, with the authors NS, JH, YF, XW, LC, to the handling editor at time of review.
